# A randomized, double-blinded, double-dummy efficacy and safety study of budesonide–formoterol Spiromax® compared to budesonide–formoterol Turbuhaler® in adults and adolescents with persistent asthma

**DOI:** 10.1186/s12890-016-0200-x

**Published:** 2016-03-17

**Authors:** J. Christian Virchow, Roberto Rodriguez-Roisin, Alberto Papi, Tushar P. Shah, Gokul Gopalan

**Affiliations:** Department of Pneumology/Intensive Care Medicine, University of Rostock, Zentrum für Innere Medizin/Medizinische Klinik 1, Rostock, Germany; Servei de Pneumologia (Institut del Tòrax), Hospital Clínic-IDIBAPS-CIBERES, Universitat de Barcelona, Barcelona, Spain; University of Ferrara, Ferrara, Italy; Teva Pharmaceuticals, Malvern, PA USA; Present address: 41 Spruce Hollow Road, Green Brook, NJ 08812 USA

**Keywords:** Asthma, Spiromax, Turbuhaler, Budesonide, Formoterol, Patient satisfaction and preference questionnaire for inhalation devices, Pulmonary, Safety

## Abstract

**Background:**

Budesonide and formoterol (BF) Spiromax® is a dry powder inhaler designed to deliver BF with maximum ease of use for patients with asthma or chronic obstructive pulmonary disease.

**Methods:**

A phase 3b, 12-week, multicenter, double-blind, double-dummy, randomized, controlled trial in patients (≥12 years) with persistent asthma. Primary objective: to demonstrate non-inferiority of twice-daily BF Spiromax 160/4.5 mcg to BF Turbuhaler® 200/6 mcg in change from baseline in weekly average of daily trough morning peak expiratory flow (PEF). Secondary endpoints included: Patient Satisfaction and Preference Questionnaire scores, change from baseline in evening PEF, trough forced expiratory volume in one second, percentage of symptom-free and rescue-free 24-hour periods, and safety.

**Results:**

The analysis was based on the per-protocol population (BF Spiromax, *n* = 290; BF Turbuhaler, *n* = 284). The least squares mean change from baseline to week 12 in morning PEF was: BF Spiromax, 18.8 L/min and BF Turbuhaler, 21.8 L/min. Non-inferiority of BF Spiromax vs BF Turbuhaler was demonstrated (the lower limit of the 95 % two-sided confidence interval was −9.02 L/min, which is greater than −15 L/min [the criteria specified for non-inferiority]). The mean difference in the total performance domains scores for BF Spiromax vs BF Turbuhaler were 0.248 at baseline and 0.353 at week 12 (both, *p* <0.001), indicating statistical superiority for BF Spiromax. No statistical or numerical differences were recorded in the total convenience domain score between the two devices. Scores for ‘device preference’ and ‘willingness to continue’ supported BF Spiromax at baseline and at week 12 (*p* = 0.0005 vs BF Turbuhaler). No significant between-group differences were observed in the other secondary efficacy endpoints. Both treatments were well tolerated, with no significant differences in adverse events or asthma exacerbations.

**Conclusions:**

This study demonstrates the non-inferiority of BF Spiromax vs BF Turbuhaler in patients (≥12 years) with asthma. More patients preferred the Spiromax device over Turbuhaler for its performance, and were willing to continue therapy with BF Spiromax beyond the 12-week study period.

**Trial registration:**

NCT01803555; February 28, 2013.

**Electronic supplementary material:**

The online version of this article (doi:10.1186/s12890-016-0200-x) contains supplementary material, which is available to authorized users.

## Background

The benefits of inhaled therapy for the treatment of chronic obstructive airway diseases including asthma and chronic obstructive pulmonary disease (COPD) are well established. Long-term controller medications, such as inhaled corticosteroids (ICS) are used with the aim of achieving daily control of asthma symptoms and preventing exacerbations. The addition of a long-acting β_2_ agonist (LABA) to ICS therapy is more effective than increasing the dose of ICS for patients with moderate-to-severe asthma who are symptomatic despite low- to medium-dose ICS [1]. The fixed-dose combination (FDC) of the ICS/LABA, budesonide–formoterol (BF) fumarate dihydrate, has shown greater improvement in pulmonary function and overall asthma control compared with either individual compound alone [2–4].

The therapeutic efficacy of an inhalation therapy such as BF requires that the drug/s reach the lower lung (specifically the smaller airways of the lungs). As such, not only the prescribed medication, but also the inhaler device plays an important role in the efficacy of drug delivery to the lungs and subsequent improvement of asthma control [5]. In the European Union, the FDC of BF is administered via a dry powder inhaler (DPI) and its approval includes use for the long-term, twice-daily, maintenance treatment of asthma. DPIs were developed with the aim of simplifying the inhalation process compared with the available pressurized metered dose inhalers (pMDIs). pMDIs require coordination with actuation; the patient needs to press down the canister and inhale the medication simultaneously. DPIs are breath-activated (most pMDIs are not), precluding the need to coordinate actuation with inhalation and may therefore be easier to use [6–8]. Although DPIs might be perceived by patients to be easier to use compared with pMDIs, many patients still have difficulties in using DPIs correctly. This may lead to loss of asthma control, or increased frequency and severity of current asthma symptoms [5]. Providing patients with the option of using a device that is easier to use may facilitate the correct handling of the inhaler and can ensure that the prescribed medication reaches the targeted areas of the lung [5].

The Spiromax device is a DPI that uses a novel X-ACT® technology [9]. The DuoResp® Spiromax formulation (BF Spiromax FDC) has been approved for use in the European Union for the treatment of adults (≥18 years old) with asthma and for patients with COPD for whom an ICS/LABA combination is indicated [10]. BF Spiromax is indicated for the long-term, twice-daily, maintenance treatment of asthma or COPD as maintenance and as an ‘as-needed’ reliever for asthma.

The bioequivalence of medium- and high-strength BF Spiromax to BF Turbuhaler was previously demonstrated [11]. Here, we present results from a phase 3b study (**A S**piromax **S**afety and **E**fficacy **T**rial [ASSET]) where the efficacy, safety and patient-reported outcomes of BF Spiromax (160/4.5 mcg [medium strength]) were compared with BF Turbuhaler (200/6 mcg [medium strength]) over 12 weeks in patients (≥12 years) with persistent asthma.

## Methods

### Study design

**Fig. 1:**
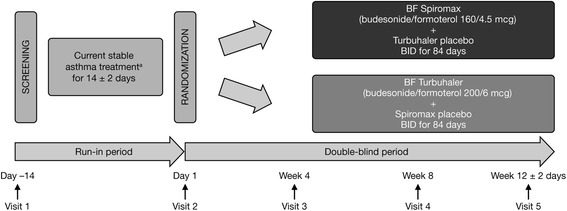
Study design. *BF* budesonide–formoterol, *BID* twice daily. ^a^Permitted asthma therapies: fluticasone propionate, beclomethasonedipropionate, budesonide, flunisolide, triamcinolone acetonide, mometasone furoate, ciclesonide

This was a randomized, double-blind, double-dummy, multicenter, parallel-group, 12-week, phase 3b study comprising a 14-day (±2 days) run-in period and a 12-week double-blind treatment period (NCT01803555/BFS-AS-306 [ASSET]; Fig. 1). The primary objective of the study was to establish the non-inferiority of BF Spiromax (160/4.5 mcg) to BF Turbuhaler (200/6 mcg) administered twice-daily for 12 weeks in patients aged ≥12 years with persistent asthma. As a secondary objective, patient preference and ease of use of the Spiromax device compared to Turbuhaler was assessed.

Independent ethics committee permission was obtained prior to commencement of the study (Additional file 1), and the study was conducted in accordance with good clinical practice and the declaration of Helsinki. All study participants, and parents/guardians of minors participating, provided signed, informed consent.

### Inclusion/exclusion criteria

Key inclusion criteria: male or female patients ≥12 years old with a diagnosis of asthma in accordance with the Global Initiative for Asthma [1], and Asthma Control Questionnaire (ACQ) score of ≥1.0 at the screening visit; persistent asthma (with a forced expiratory volume in 1 s [FEV_1_] of 40–85 % of the predicted value, as per the National Health and Nutrition Examination Survey III reference values) for ≥3 months that had been stable for at least 30 days before the screening visit; a ≥12 % reversibility of FEV_1_ within 30 min after two to four inhalations of salbutamol/albuterol; or documented ≥12 % reversibility of FEV_1_ (and 200 mL increase) within the last 12 months. Any prior or concomitant therapy, medication or procedure a patient took within 90 days prior to study drug administration, or during the study period, was documented by the investigator. Patients must have received a short-acting β_2_ agonist (SABA) and an ICS for a minimum of 8 weeks before the screening visit, have been maintained on a stable dose of ICS for 4 weeks before the screening visit, and must have been able to replace their current SABA with salbutamol/albuterol for use as needed throughout the study.

Key exclusion criteria included: a history of life-threatening asthma; actual or suspected bacterial or viral infection of the upper or lower respiratory tract, sinus, or middle ear infection within the last 2 weeks prior to screening; an asthma exacerbation requiring oral corticosteroids within 1 month prior to the screening visit. The use of oral or depot corticosteroids within 4 weeks before the screening visit and the use of tobacco products within the past 12 months, or a smoking history of ≥10 pack years were prohibited. In addition, patients were asked to not engage in strenuous exercise and to avoid cold air exposure on the mornings of scheduled clinic visits.

### Study treatments

Patients, enrolled from outpatient clinics (at hospital or private pulmonary specialist practices; the study took place from July 2013 to March 2014), were randomly assigned (1:1) to receive one of two treatments taken by two inhalations, twice daily for 12 weeks (Fig. 1): BF Spiromax (160/4.5 mcg) plus placebo Turbuhaler − the Spiromax group, or BF Turbuhaler (200/6 mcg) plus placebo Spiromax − the Turbuhaler group.

Patients were randomly assigned to treatment through a qualified randomization service provider (interactive response technology [IRT]). This system was used to ensure a balance across treatment groups; no effort was made to maintain a balance among treatment groups within an investigational center. Patients and materials number were centrally randomized and distributed using the IRT. The randomization code was generated by the Clinical Supply Chain (CSC) department following specifications from the Biostatistics Department. A statistician not assigned to the study was responsible for reviewing the randomization code, and the final randomization code was maintained by the CSC department. BF Spiromax and placebo Spiromax were presented in a Spiromax inhalation device, contained in a sealed foil pouch, and packed in an individual carton. Similarly, BF Turbuhaler and placebo Turbuhaler were presented in a Turbuhaler inhalation device and packed in an individual carton. To maintain the study blinded there was no discernible differences between BF Spiromax and placebo Spiromax or BF Turbuhaler and placebo Turbuhaler; both were identically labeled for each inhaler. Patients in each arm randomly chose which device they used first (placebo or active).

### Study assessments

Lung function was measured by spirometry tests. Patients were required to record daily morning (ante meridiem [AM]) and evening (post meridiem [PM]) asthma symptom scores, AM and PM peak expiratory flow (PEF) values, and medication administration in the paper diaries provided. The asthma symptom scores were recorded on a linear scale from 0 to 5 (PM assessment) where 0 = no symptoms during the day and 5 = symptoms so severe that the patient could not go to work or perform normal daily activities, and from 0 to 4 (AM assessment) where 0 = no symptoms during the night and 4 = symptoms so severe that the patient did not sleep at all. The PEF was measured twice-daily by the patient using a hand-held electronic peak flow meter. Peak flow measurements were performed at each of the participating centers using the center’s own equipment, which were calibrated daily. Three measurements were taken and the highest value was recorded (the two largest FEV_1_ values had to be within 150 mL of each other). The best result, according to the American Thoracic Society/European Respiratory Society criteria [12], was recorded and used in the analysis.

The pulmonary function tests (FEV_1_ and PEF) were measured in the morning by spirometry at the clinic visits within ± 1 h of the time of the spirometry testing at the screening visit.

Patients completed the ACQ [13] at each visit to provide an assessment of clinical impairment due to asthma and any change in asthma control. Additionally, the Patient Satisfaction and Preference Questionnaire for Inhalation Devices (PASAPQ^©^ [Copyright by Boehringer Ingelheim International GmbH 2004]) [14] and a question on satisfaction with the overall quickness of using the inhaler were completed for each device at baseline (day 1 of week 1 of the double-blind treatment period) and at the end of the study. The Asthma Quality of Life Questionnaire (AQLQ) [15, 16] was completed at baseline and at subsequent visits.

Patient compliance to administration of study medication was assessed at each visit by reviewing patient diaries for completion of the study procedure entries and by dose counter readings on the inhaler devices.

Serial spirometry, with pulmonary function (FEV_1_ and PEF) tests being performed from 30 min before dosing to 3 h after dosing, was conducted in a planned subset of 30 patients from three to four sites at the baseline visit (visit 2). Drug safety was monitored throughout the study by recording of adverse events (AEs) by the investigator (all reported or observed signs and symptoms were recorded individually, except when considered manifestations of a medical condition or disease state). When such a diagnosis was made, this was recorded collectively as a single diagnosis in the case report form provided and, for serious AEs (SAEs), this was recorded on the Serious Adverse Event Transmittal Form. Measurement of vital signs and oropharyngeal examination for candidiasis, clinical laboratory testing (blood eosinophil count and serum human chorionic gonadotropin levels [as appropriate]) was conducted at screening and at the final clinic visit.

### Endpoints

The primary efficacy endpoint for this study was the change from baseline in weekly average of daily trough (pre-dose and pre-rescue bronchodilator) AM PEF over the 12-week treatment period. Secondary efficacy endpoints were: PASAPQ scores and inhaler quickness satisfaction score at week 12, the change from baseline in weekly average of daily PM PEF, trough (AM pre-dose and pre-rescue bronchodilator) FEV_1_ over the 12-week treatment period, and percentage of symptom-free and rescue-free 24-hour periods during the 12-week treatment period.

### Statistical analysis

The sample size and power calculations were based on the non-inferiority comparison of change from baseline in weekly average of daily trough AM PEF between BF Spiromax and BF Turbuhaler. Two hundred and seventy patients per treatment group (a total of 540 patients) were required to provide an approximate statistical power of 90 % at a significance level of 0.025 for the one-sided non-inferiority test. Non-inferiority was defined as the lower limit of the two-sided 95 % confidence interval (CI) for the treatment difference in change from baseline in the weekly average of daily trough AM PEF over the 12-week treatment period greater than −15 L/min.

The intent-to-treat population included all randomized subjects. The per-protocol (PP) population included data from all randomized subjects obtained before experiencing major protocol deviations/violations. The efficacy analyses were conducted on the PP population. The safety population included all randomized subjects who received at least one dose of study medication.

The change from baseline in weekly average of daily trough AM and PM PEF over the 12-week treatment period was performed using a repeated measures mixed model with adjustment for baseline weekly average of daily trough AM or PM PEF, sex, age, treatment, time and treatment-by-time interaction.

The change from baseline in the percentage of symptom-free 24-hour periods and the change from baseline in the percentage of rescue-free 24-hour periods, during the 12-week treatment period, were analyzed using the Wilcoxon–Mann–Whitney test.

The change from baseline in trough (AM pre-dose and pre-rescue bronchodilator) FEV_1_ over the 12-week treatment period were analyzed using a repeated measures mixed model with effects due to baseline trough FEV_1_, gender, age, visit, treatment, and visit-by-treatment interaction.

Total and individual satisfaction scores (PASAPQ performance and convenience domain scores at baseline and at week 12) were analyzed using the paired *t*-test.

The change from baseline in the device preference category score, overall inhaler quickness satisfaction score, and the ‘willingness to continue’ using the device score at week 12 were analyzed using an analysis of covariance (ANCOVA) model.

## Results

### Study population

**Fig. 2:**
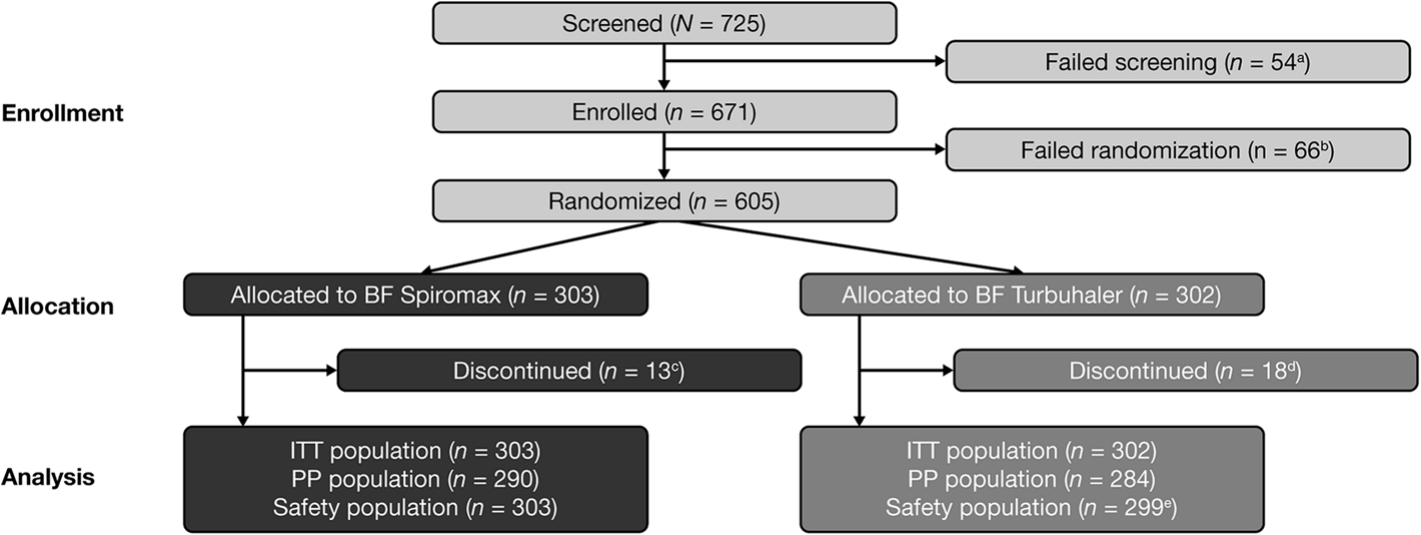
Patient disposition. *AE* adverse event, *BF* budesonide–formoterol, *ITT* intent-to-treat, *PP* per protocol. ^a^Inclusion criteria not met, *n* = 25; exclusion criteria met, *n* = 6; withdrawal by patient, *n* = 6; AEs, *n* = 1; other, *n* = 16. ^b^Inclusion criteria not met, *n* = 5; withdrawal by patient, *n* = 5; randomization criteria not met, *n* = 2; AE, *n* = 1; lost to follow-up, *n* = 1; other, *n* = 52. ^c^Protocol violation, *n* = 3; withdrawal by subject, *n* = 3; AE, *n* = 2; asthma exacerbation, *n* = 1; non-compliance, *n* = 1; other, *n* = 3. ^d^Protocol violation, *n* = 5; withdrawal by subject, *n* = 3; AE, *n* = 2; lost to follow-up, *n* = 2; non-compliance, *n* = 1; other, *n* = 5. ^e^Three patients who were randomized to the BF Turbuhaler 200/6 mcg did not receive treatment (due to randomization error, ‘withdrawal by subject’, ‘other’ reasons; each, *n* = 1)

Patients were enrolled from out-patient clinics (part of a hospital or from pulmonary specialist private practices; the study tool place from July 2013 to March 2014). A total of 605 patients were randomized: 303 in the BF Spiromax group and 302 in the BF Turbuhaler group. Three patients who were randomized to the BF Turbuhaler 200/6 mcg did not receive treatment (due to randomization error, ‘withdrawal by subject’, and ‘other’ reason, each *n* = 1). The safety population included 602 patients, and the PP population included 574 patients (Fig. 2). Compliance to the study medication in the safety population was 97.71 % in the BF Spiromax group and 97.49 % in the BF Turbuhaler group. Patient demographics per treatment group are shown in Table 1. The median age of patients were 48 and 47 years for the BF Spiromax and Turbuhaler groups, respectively; 43 and 47 % were male, and 98 and 99 % were white. There were no significant/statistical differences between the two groups across all parameters assessed in Table 1.

**Table 1:** Summary of baseline characteristics and demographic data (ITT population)

	BF Spiromax (*n* = 303)	BF Turbuhaler (*n* = 302)
Age, years (mean ± SD)	48.1 ± 16.24	46.9 ± 16.89
Sex, *n* (%)		
Male	131 (43)	141 (47)
Female	172 (57)	161 (53)
Race, *n* (%)		
White	297 (98)	300 (>99)
Black	2 (<1)	1 (<1)
Asian	1 (<1)	1 (<1)
Other	3 (<1)	0 (0)
Weight, Kg (mean ± SD)	77.4 ± 17.25	78.6 ± 17.88
Median weekly averaged AM PEF, L/min (range)	318.6 (82.9–632.9)	345.7 (108.6–604.3)
Median weekly averaged PM PEF, L/min (range) (L/min)	328.6 (98.6–650.0)	352.9 (127.1–635.7)
Median baseline FEV_1_, L (range) (L)	2.1 (0.9–4.2)	2.3 (0.8–4.0)
Prior asthma medications, ≥5 % (*n* [%])		
Salbutamol	290 (96)	281 (93)
Budesonide	145 (48)	139 (46)
Fluticasone	77 (25)	73 (24)
Budesonide–formoterol	48 (16)	43 (14)
Fluticasone and salmeterol	43 (14)	37 (12)
Average symptom-free 24-hour period, % (mean ± SD)	19 ± 31.67^a^	15.3 ± 26.4^b^
Average rescue-free 24-hour period, % (mean ± SD)	33.3 ± 38.26^a^	30.9 ± 37.02^b^

*AM* ante meridiem (morning), *BF* budesonide–formoterol, *FEV*_*1*_ forced expiratory volume in 1 s, *ITT* intent-to-treat, *PEF* peak expiratory flow, *PM* post meridiem (evening), *SD* standard deviation

^a^ *n* = 290 (per-protocol population)

^b^ *n* = 284 (per-protocol population)

### Pulmonary function tests

**Fig. 3:**
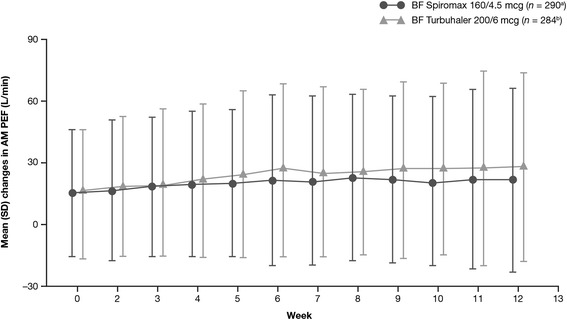
Mean change in average weekly morning PEF. *AM* ante meridiem (morning), *BF* budesonide–formoterol, *PEF* peak expiratory flow, *SD* standard deviation. Data shown are mean change from baseline on weekly average values. Error bars represent SD. ^a^ *n* = 290 at baseline; dropped to *n* = 263 by week 12. ^b^ *n* = 284 at baseline; dropped to *n* = 256 by week 12

As shown in Fig. 3, the mean change from baseline in the weekly average of daily trough AM PEF, the primary endpoint for this study, was 18.8 L/min for BF Spiromax and 21.8 L/min for BF Turbuhaler (*p* = 0.3387). The lower bound of the two-sided 95 % CI for the treatment difference was −9.02 L/min, which is greater than −15.0 L/min, the criteria specified for non-inferiority for this study. Therefore, non-inferiority of BF Spiromax (160/4.5 mcg) vs BF Turbuhaler (200/6mcg) has been achieved. In both treatment groups, there was an improvement in the weekly average of daily trough AM PEF over the 12-week treatment period (Fig. 3).

**Fig. 4:**
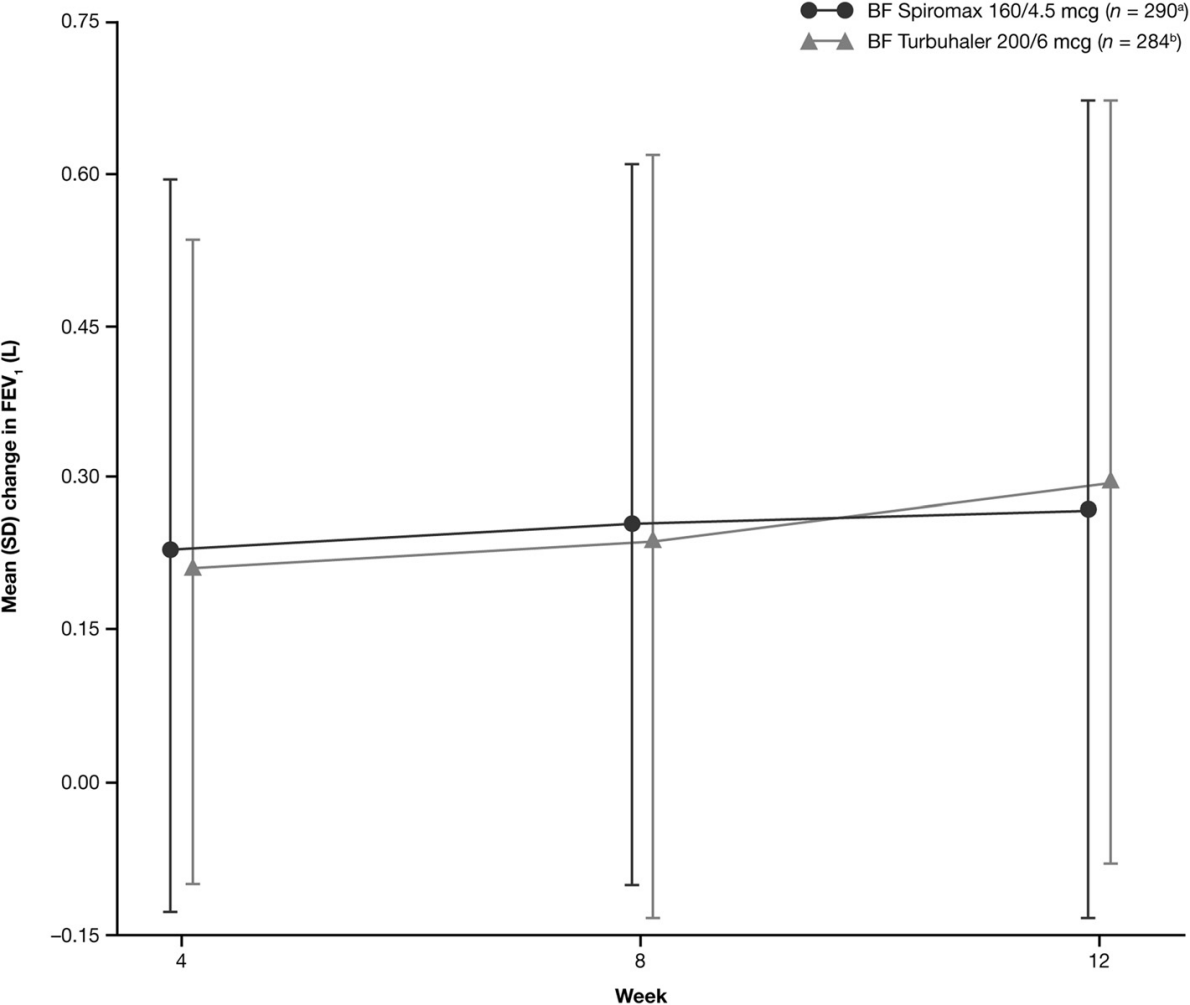
Mean change in trough FEV_1_. *BF* budesonide–formoterol, *FEV*_*1*_ forced expiratory volume in 1 s, *SD* standard deviation. ^a^ *n* = 290 at baseline; dropped to *n* = 276 by week 12. ^b^ *n* = 284 at baseline, dropped to *n* = 264 by week 12

The mean change from baseline in the weekly average of daily trough PM PEF and the least squares mean changes in FEV_1_ over the 12-week treatment period was also similar for both treatment groups. The change in daily trough PM PEF was 18.661 L/min for BF Spiromax and 21.740 L/min for BF Turbuhaler (95 % CI: −8.82, 2.67; *p* = 0.2930). In both treatment groups, an improvement in weekly average of daily trough PM PEF was noted. The least squares mean changes in FEV_1_ from baseline were 0.325 and 0.318 L for BF Spiromax and BF Turbuhaler, respectively (95 % CI: −0.04, 0.06; *p* = 0.7661; Fig. 4).

### Serial spirometry – subset analysis

Following the first dose, more patients with BF Spiromax vs BF Turbuhaler had an increase of ≥12 % above their baseline FEV_1_ for up to 6 h (16 [94 %] vs 12 [71 %] patients, respectively). Of those patients who had a ≥12 % increase in FEV_1_, there was no notable difference in the onset time when this increase occurred (0.6 and 0.9 h in the BF Spiromax vs BF Turbuhaler groups, respectively). The duration of the effect was comparable between the BF Spiromax and the BF Turbuhaler (4.4 vs 3.8 h with BF Spiromax vs BF Turbuhaler, respectively).

### Asthma symptoms and rescue medication

When patients were assessed for their asthma symptoms and rescue medications use, no significant differences were observed between the two treatment groups. The change from baseline to week 12 in the mean percentage of symptom-free 24-hour periods was 30.0 % for the BF Spiromax and 32.5 % for BF Turbuhaler (*p* = 0.3082). The change in percentage of rescue-free 24-hour periods from baseline to week 12 was also similar for both treatment groups: BF Spiromax 37.8 %, BF Turbuhaler 40.2 % (*p* = 0.4300). There was an increase from baseline in the mean percentage of rescue-free 24-hour periods in both treatment groups (baseline/week 12: 33.3 %/71 % BF Spiromax and 30.9 %/71.1 % BF Turbuhaler). There were no significant differences between ACQ and AQLQ scores with BF Spiromax compared with BF Turbuhaler over the 12-week treatment period.

### Patient satisfaction and preference

**Fig. 5:**
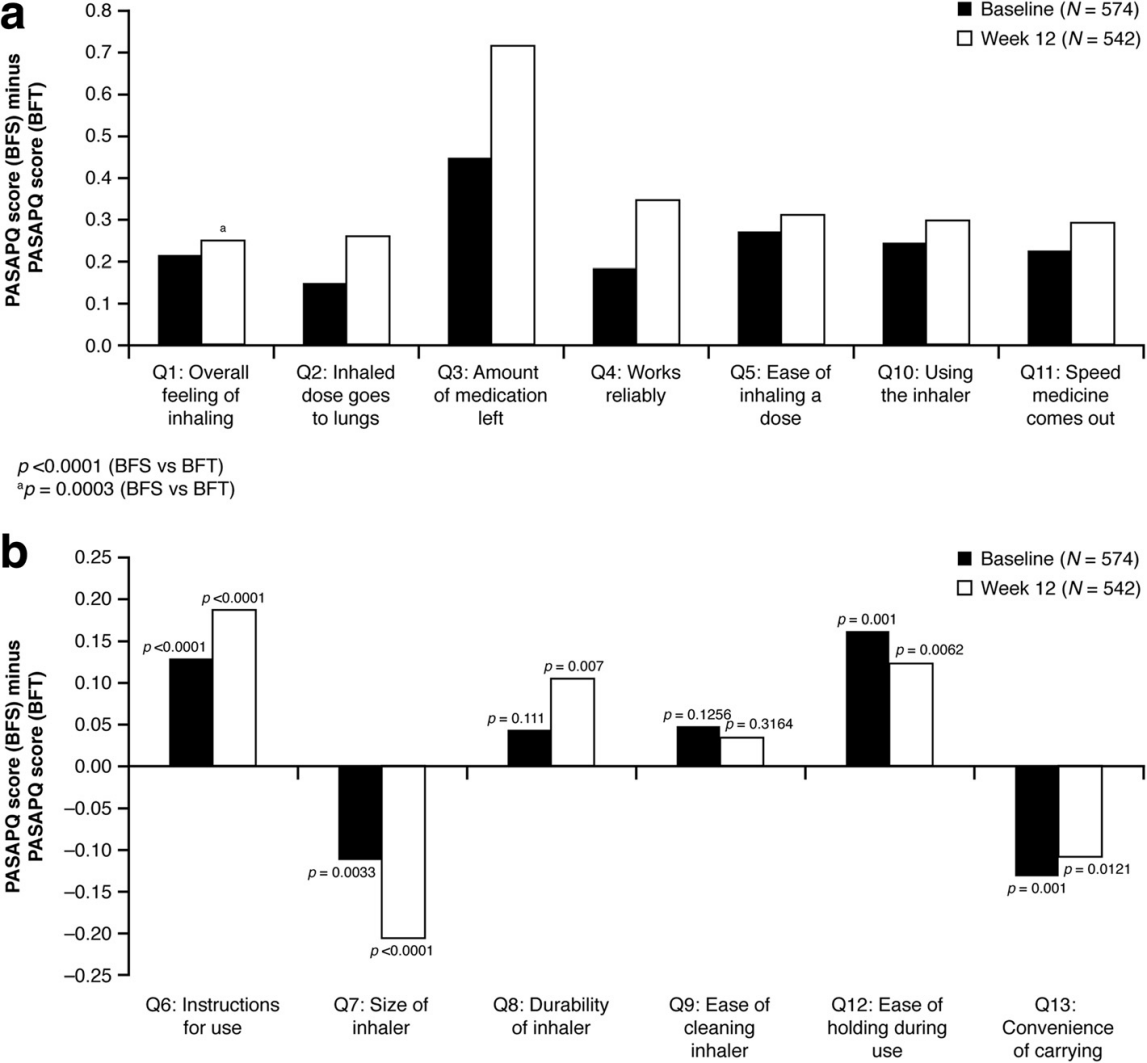
Differences in mean Spiromax PASAPQ scores vs mean Turbuhaler PASAPQ scores at baseline^*^ and week 12^*^ (**a**) performance domains (**b**) convenience domains. *BFT* budesonide–formoterol Turbuhaler, *BFS* budesonide–formoterol Spiromax, *PASAPQ* Patient Satisfaction and Preference Questionnaire for Inhalation Devices. Positive Y-axis values indicate that Spiromax performed better than Turbuhaler; negative Y-axis values indicate that Turbuhaler performed better than Spiromax

**Fig. 6:**
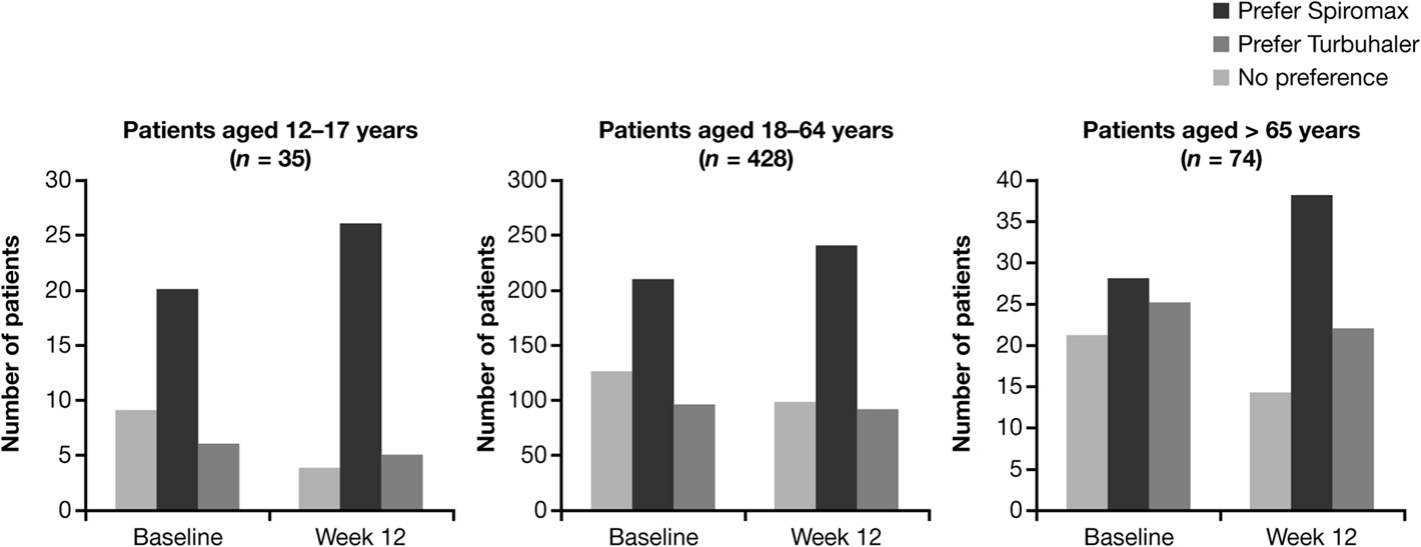
Device preference by age group at baseline and week 12

In general, the differences between the groups in PASAPQ domains scores were small. The mean difference in the total performance domains scores for BF Spiromax vs BF Turbuhaler were 0.248 and 0.353 at baseline and week 12, respectively (both, *p* <0.001), indicating statistical superiority for the Spiromax device in the total performance domains scores. Similar findings were recorded when comparing the mean differences in individual performance domains scores (PASAPQ Q1−Q5, Q10, and Q11) for Spiromax vs Turbuhaler. The Spiromax device was superior to the Turbuhaler device at baseline and week 12 for each of the individual performance domains scores (the value for Spiromax PASAPQ performance domain score minus Turbuhaler PASAPQ performance domain score was in the positive range; *p* ˂0.0001 [except for Q1, *p* = 0.0003]; Fig. 5a). The mean differences in the total convenience domains scores for Spiromax vs Turbuhaler were 0.023 (*p* = 0.3432) at baseline and 0.023 (*p* = 0.4190) at week 12, indicating no statistical or numerical differences between the two devices in the total convenience domain scores. An analysis of the individual components of the convenience domains scores (PASAPQ Q6−Q9, Q12−Q13; Fig. 5b) between the two devices revealed that the Spiromax device was superior to Turbuhaler (the value for Spiromax PASAPQ convenience domain score minus Turbuhaler PASAPQ convenience domain score was in the positive range) at baseline and week 12 for Q6 (instruction for use), Q8 (durability of the inhaler), Q9 (ease of cleaning the inhaler) and Q12 (ease of holding during use). Conversely, Turbuhaler was superior to Spiromax (the value for Spiromax PASAPQ convenience domain score minus Turbuhaler PASAPQ convenience domain score was in the negative range) at baseline and week 12 for Q7 (size of the inhaler) and Q13 (convenience of carrying) of the convenience domains scores (Fig. 5b). The change in PASAPQ ‘willingness to continue’ use score from baseline to week 12 was significantly greater for the Spiromax device than for Turbuhaler (3.65 vs −3.951; *p* = 0.0005). Device preference was higher for the Spiromax device vs the Turbuhaler device at baseline and week 12. At baseline, 256 patients preferred the Spiromax device vs 126 patients who preferred Turbuhaler (*p* <0.0001). At week 12, 304 patients preferred the Spiromax device vs 118 who preferred the Turbuhaler device (*p* <0.0001). The device preference for the Spiromax vs the Turbuhaler was maintained when the population was evaluated according to age group (Fig. 6). The change in overall inhaler quickness satisfaction from baseline to week 12 was significantly greater for the BF the Spiromax than for the BF Turbuhaler (0.316 vs −0.012; *p* = 0.0005).

### Safety

The incidence of AEs was similar between the two treatment groups; at least one AE was reported by 39 % of patients in the BF Spiromax group and by 35 % in the BF Turbuhaler group. Details of these AEs are shown in Table 2. Nasopharyngitis and headache were the most common AEs reported in both study groups. SAEs were experienced by <1 % of patients in the BF Spiromax group and by 2 % in the BF Turbuhaler group. There were four SAEs, all of which were deemed unrelated to the study intervention by the investigator: one patient (<1 %) in the BF Spiromax study group experienced pneumonia and three patients (1 %) in the Turbuhaler study group experienced a bradycardia episode leading to discontinuation, an intervertebral disc protrusion, and a supraventricular tachycardia (*n* = 1 each).

**Table 2:** AEs in ≥2 % of patients (safety population)

	Number (%) patients		
	BF Spiromax 160/4.5 mcg	BF Turbuhaler 200/6 mcg	Total
	(*n* = 303)	(*n* = 299)	(*N* = 602)
Patients with at least one AE	117 (39)	106 (35)	223 (37)
Infections and infestations	61 (20)	69 (23)	130 (22)
Nasopharyngitis	31 (10)	25 (8)	56 (9)
Rhinitis	6 (2)	7 (2)	13 (2)
Bronchitis	3 (<1)	7 (2)	10 (2)
Respiratory, thoracic and mediastinal disorders	33 (11)	22 (7)	55 (9)
Cough	11 (4)	8 (3)	19 (3)
Oropharyngeal pain	7 (2)	5 (2)	12 (2)
Dysphonia	9 (3)	1 (<1)	10 (2)
Nervous system disorders	21 (7)	26 (9)	47 (8)
Headache	18 (6)	24 (8)	42 (7)
Gastrointestinal disorders	18 (6)	14 (5)	32 (5)
General disorders and administration site conditions	9 (3)	4 (1)	13 (2)

*AE* adverse event, *BF* budesonide–formoterol

Two patients in each study group discontinued treatment due to AEs. In the BF Spiromax group, one patient discontinued treatment due to bacterial respiratory tract infection and another patient discontinued treatment due to dyspnea. In the BF Turbuhaler group, one patient discontinued treatment due to nasopharyngitis, dyspnea, oropharyngeal pain, and rhonchi and a second patient discontinued treatment due to serious bradycardia (reported by the same patient mentioned above in the SAE section). There were no deaths reported in this study.

## Discussion

ASSET is the first clinical study designed to compare the efficacy and safety of BF Spiromax with BF Turbuhaler in adult and adolescent patients with persistent asthma. It demonstrated that BF Spiromax was non-inferior to treatment with BF Turbuhaler with respect to the mean change from baseline in the weekly average of daily trough AM PEF (the relationship between the variability of the PEF 20 L/Min and the non-inferiority reference value 15 L/Min was based on a non-inferiority limit of a value equating to a third of the assumed standard deviation of the PEF at any given time point, which was set at 45 L/min). Similar improvements in FEV_1_ were achieved with both devices.

BF administered in a FDC is an established and effective therapy with a long-standing, good safety profile for the treatment of asthma [17] and COPD [4, 18]. In our study, BF Spiromax showed similar efficacy (lung function and symptom control) and safety compared to BF Turbuhaler over the 12-week treatment period. Furthermore, serial spirometry in a subset of patients performed to test and compare the formoterol portion of the combination showed that the time to onset (increase [from baseline] in FEV_1_ of ≥12 %) and the duration of this effect were similar between the devices, further supporting the therapeutic equivalence of BF Spiromax to BF Turbuhaler.

Although the efficacy and safety of a medication are important considerations when selecting a treatment for asthma or COPD, the effectiveness of therapy is also dependent on the patient using their inhaler correctly and as prescribed every time. In addition, the choice of an inhaler device and the patient’s opinion on a particular device are also important factors in asthma management [1]. However, many patients with asthma have poor adherence to therapy. According to the medication adherence taxonomy recommended by Vrijens et al. [19], poor or non-adherence in the context of patients with asthma is one or a combination of the following: (1) not taking medication at the correct time (example: twice-daily regimens – patient should normally separate each dose by ~12 h) or late in taking the treatment as prescribed (e.g. ‘missing a dose’); (2) suboptimal implementation of the dosing regimen (e.g. not using the inhaler correctly); and (3) early discontinuation of treatment (e.g. the patient stops taking the medication) [19]. In asthma, poor adherence can lead to loss of asthma control and/or worsening of asthma symptoms [1, 20]. The ultimate goal of treatment is optimal pharmacotherapy due to its implicit association with optimal clinical outcomes – this requires good medication adherence [19]. An inhaler that is easy to use, carry, and store, and that is reliable and intuitive might improve patients’ adherence.

Compared to the Turbuhaler device, which requires a number of steps for actuation (removing the cap, holding the inhaler in an upright position and turning the grip as far as it will go and back), the Spiromax device has the theoretical advantage of being more intuitive and easier to use as it can be ready for actuation after the single step of opening the cap. Therefore, in this study, patients were asked to provide feedback with regards to the performance and convenience of the Spiromax and Turbuhaler devices (at baseline and after 12 weeks) using the validated PASAPQ. The outcomes from the PASAPQ analysis indicate that Spiromax was superior to Turbuhaler for all performance domain questions asked. However, no statistical or numerical differences were observed between the two devices when comparing the total convenience scores. When assessing the individual device convenience scores, Spiromax was also superior to Turbuhaler at baseline and week 12 for its ‘instruction for use’, ‘durability of the inhaler’, ‘ease of cleaning’, and ‘ease of holding during use’, which might be related to the reduced number of steps required up to the point when actuation of the BF dose from Spiromax can occur. On the other hand, the Turbuhaler was deemed superior to Spiromax for its size and ‘convenience of carrying’. Patients were more willing to ‘continue use’ of the Spiromax device compared with Turbuhaler (after the 12-week treatment period) and reported ‘preference’ for the Spiromax device over the Turbuhaler. These results might reflect the more intuitive handling of Spiromax and could have positive implications for future adherence to treatment.

Among the strengths of this study is the assessment of patient preference via the validated PASAPQ in addition to assessment of clinical efficacy. Not all phase 3 controlled studies consider patients’ opinions and instead focus on clinical efficacy and safety only. The ASSET study was conducted to reassure physicians and other healthcare professionals (HCPs) of the practicability of Spiromax in terms of its patient-reported ‘greater ease of use’ (preference, ‘willingness to continue’ use and performance) and its similarity to BF Turbuhaler in clinical efficacy and safety (incidence/frequency of AEs/vital signs, etc.). However, it should be noted that these data were collected as part of a phase 3, controlled, clinical trial in patients with asthma, which limits the transferability of the results to everyday practice. In the ‘real world’, the asthma population is more heterogeneous and disease monitoring is according to physicians’ routine clinical practice (vs a clinical trial setting).

Ultimately, real-world effectiveness trials will be needed to determine if these improvements can lead to improved adherence and clinical outcomes. Data from real-world studies where patients would be randomly allocated to receive treatment with either Spiromax or Turbuhaler would be of particular interest in the context of this study. Such real-word effectiveness studies are required to determine whether improvements based on patients’ preference and satisfaction with the Spiromax inhaler would lead to improved adherence and clinical outcomes in real-world scenarios. Detailed patient and HCP opinion regarding the ‘ease of use’ of the Spiromax device vs Turbuhaler and also the proportion of patients achieving mastery (absence of observed errors) in Spiromax/Turbuhaler inhaler technique are being assessed in separate studies [21].

## Conclusions

BF Spiromax 160/4.5 mcg was shown to be similar to BF Turbuhaler 200/6 mcg in relation to efficacy and safety in the treatment of asthma; however, specific domains of the PASAPQ showed greater benefits with Spiromax over Turbuhaler. The PASAPQ analysis suggests that Spiromax was superior to Turbuhaler for all the performance domains. No differences (statistical or numerical) were observed when comparing the total convenience scores for Spiromax and Turbuhaler. Patients preferred the Spiromax over the Turbuhaler and considered it easier to use. Studies looking to substantiate patient preference, inhaler ease of use and inhaler handling errors (critical and non-critical errors) comparing BF Spiromax and Turbuhaler are currently ongoing and will clarify if the benefits observed in this trial are repeated. Ultimately, real-world effectiveness trials will be needed to determine if these improvements can lead to improved adherence and clinical outcomes.

## Additional file

Additional file 1: List of Independent Ethics Committees and Institutional Review Boards. (DOCX 45 kb)

## Abbreviations

ACQ: asthma control questionnaire
AE: adverse event
AM: ante meridiem (morning)
AQLQ: asthma quality of life questionnaire
ASSET: a spiromax safety and efficacy trial
BF: budesonide–formoterol
BFS: budesonide–formoterol Spiromax
BFT: budesonide–formoterol Turbuhaler
CI: confidence interval
COPD: chronic obstructive pulmonary disease
CSC: clinical supply chain
DPI: dry powder inhaler
FDC: fixed-dose combination
FEV_1_: forced expiratory volume in 1 s
HCP: healthcare professional
ICS: inhaled corticosteroid
IRT: interactive response technology
ITT: intent-to-treat
LABA: long-acting ß_2_ agonist
PASAPQ: patient satisfaction and preference questionnaire for inhalation devices
PEF: peak expiratory flow
PM: post meridiem (evening)
pMDIs: pressurized metered dose inhalers
PP: per-protocol
SD: standard deviation
SABA: short-acting ß_2_ agonist
SAE: serious adverse event
