# Supplementary material for: A randomized, double-blinded, double-dummy efficacy and safety study of budesonide–formoterol Spiromax® compared to budesonide–formoterol Turbuhaler® in adults and adolescents with persistent asthma
Source: BMC Pulm Med. 2016 Mar 17;16:42. doi: 10.1186/s12890-016-0200-x (PMC4794916; doi:10.1186/s12890-016-0200-x)
Supplement: Additional file 1: — List of Independent Ethics Committees and Institutional Review Boards. (DOCX 45 kb) [file 12890_2016_200_MOESM1_ESM.docx]

**List of Independent Ethics Committees and Institutional Review Boards**

| **Center number** | **Investigator** | **IEC/IRB name Committee chairperson**a | **Address** | **Country** |
| --- | --- | --- | --- | --- |
| 33018  33019  33020 | Forstner, Bernhard  Würtz, Josef  Würtz, Peter | Ethikkommission des Landes  Oberösterreich  Chairperson: Prof. Johannes  Fischer | Linz | Austria |
| 37029  37030  37031 | De Meulemeester, Marc  Martinot, Jean- Benoit Vereecken, Guy | Central EC  Secrétaire du Comité d’éthique  Chairperson: Ms Stephanie Ellis | Bruxelles | Belgium |
| 54058 | MUDr. Kopecká, Daniela | Etická komise FN u sv. Anny v  Brně  Chairperson: doc. MUDr. Vladimír Soška, CSc | Brno | Czech  Republic |
| 54059 | MUDr. Mareš, Jaroslav | Etická komise FN Hradec  Králové  Chairperson: MUDr. Jiří Vortel | Králové | Czech  Republic |
| 54061 | MUDr. Novosad, Libor, Ph.D. | Etická komise FN Hradec  Králové  Chairperson: MUDr. Jiří Vortel | Králové | Czech  Republic |

| **Center number** | **Investigator** | **IEC/IRB name Committee chairperson**a **Assurance number** | **Address** | **Country** |
| --- | --- | --- | --- | --- |
| 54063 | MUDr. Škopková, Olga | Etická komise Vítkovické nemocnice, a.s.  Chairperson: MUDr. Vladimír  Čížek | Ostrava - Vítkovice | Czech  Republic |
| 54064 | MUDr. Veverka, Josef | Lokální Etická komise FN Plzeň  Chairperson: Doc. MUDr. Jindřich Fínek, Ph.D. | Plzeň | Czech  Republic |
| 54065 | MUDr. Seberová, Ester | Lokální Etická komise FN Plzeň  Chairperson: Doc. MUDr. Jindřich Fínek, Ph.D. | Plzeň | Czech  Republic |
| 39020  39021 | Backer, Vibeke  Vestbo, Jorgen | The Scientific Ethical Committee B for Denmark Capital Region  Chairperson: Mr. Mikael Bitsch | Hillerød | Denmark |
| 40001  40002  40003  40004 | Karjalainen, Jussi Nieminen, Eija Elo, Juhani Hakulinen, Auli | Ethics Committee Varsianais- Suomi Hospital District Administrative Centre/Research Office  Chairperson: Prof. Mika  Scheinin | Turku | Finland |

| **Center number** | **Investigator** | **IEC/IRB name Committee chairperson**a **Assurance number** | **Address** | **Country** |
| --- | --- | --- | --- | --- |
| 35088  35089  35090  35091  35092  35093 | Leroyer, Christophe Tardif, Philippe Boye, Alain  Verdier, Serge Marty, Jacques Devouassoux, Gilles | Comité de Protection des  Personnes – Ouest III  Chairperson: Mr P. THOMAS | Poitiers Cedex | France |
| 32252  32241  32253  32240  32258  32251  32256  32244  32255  32257  32243  32249  32247  32250  32259  32246  32254 | Käßner, Frank MD Beck, Ekkehard MD Keller, Claus MD Ballenberger,  So tSabine Scholz, Gerhard Kardos, Peter Schenkenberger, Isabelle Gebhardt, Rainer MD  Linnhoff, Anneliese  MD Schmidtmann, Sören  Förster, Karin Jandl, Margret MD Hofbauer, Peter MD Kanniess, Frank MD Zachgo, Wolfgang MD  Geßner, Christian  MD  Lindemann, Ludger  MD | Landesärztekammer  Brandenburg  Chairperson: Prof. Dr. med. Michael Matthias | Cottbus | Germany |
| 51065  51067  51068  51070  51072  51073  51074  51075  51076  51077 | Bálint, Beatrix Meszaros, Imre Schlezak, Judit Szekely, Gyongyi Timar, Mihaly Laki, Istvan  Mark, Zsuzsanna Gomori, Katalin Bocskei, Csaba Juhasz, Gabor | Egészségügyi Tudományos Tanács, Klinikai Farmakológiai Etikai Bizottsága  Chairperson: Prof. Dr. Fürst  Zsuzsanna | Budapest, | Hungary |

| **Center number** | **Investigator** | **IEC/IRB name Committee chairperson**a **Assurance number** | **Address** | **Country** |
| --- | --- | --- | --- | --- |
| 80035 | Adir Yochai | Helsinki Committee, Carmel MC Chairperson: Dr. Ruth Kitzes  Assurance Number : N/A | Haifa | Israel |
| 80038 | Fink, Gershon | Helsinki Committee, Kaplan  MC  Chairperson: Dr. Lidia Harcavi | Rehovot | Israel |
| 80039 | Kramer, Mordechai  Reuben | Helsinki Committee, Rabin MC, Beilinson Campus  Chairperson: Prof. Meir Lahav | Petach Tikvah | Israel |
| 80040 | Shitrit, David | Helsinki Committee, Meir MC Chairperson: Prof. Ilan Cohen | Kfar Sab | Israel |
| 80041 | Stav, David | Helsinki Committee, Assaf  Harofeh MC  Chairperson: Prof. Eitan Scapa | Zerifin | Israel |
| 30055 | Paggiaro Pierluigi,  <MD> | Comitato Etico Area Vasta Nord Ovest Sezione Autonoma del Comitato Etico Regionale per la Sperimentazione Clinica  Chairperson: Mr. Romano  Danesi | Pisa | Italy |

| **Center number** | **Investigator** | **IEC/IRB name Committee chairperson**a **Assurance number** | **Address** | **Country** |
| --- | --- | --- | --- | --- |
| 38049  38048 | Pasma, Henk  Boersma, Wim | METC Noord Holland  Chairperson: Dr. G.W. ten  Tusscher | Alkmaar | The  Netherlands |
| 53099  53100  53101  53102  53103  53104  53105  53106  53107  53109  53110  53111  53113  53114  53115  53116  53119  53120 | Jutel, Marek Jasieniak-Pinis, Grazyna Olszewska-Ziaber, Agnieszka  Emeryk, Andrzej Dymek, Lucyna Malosek, Dorota Majorek-  Olechowska,  Bernadetta  Gross-Tyrkin, Ewa Kuna, Piotr Dyczek, Andrzej Ploszczuk, Anna Pulka, Grazyna Jarzab, Jerzy  Bodzenta-Lukaszyk, Anna  Fal, Andrzej Hofman, Teresa Waszkuc Golonko, Joanna  Dobek, Rafal | Komisja Bioetyczna przy  Dolnośląskiej Izbie Lekarskiej  Chairperson: Lech Zynda MD, Phd | Wrocław | Poland |

| **Center number** | **Investigator** | **IEC/IRB name Committee chairperson**a **Assurance number** | **Address** | **Country** |
| --- | --- | --- | --- | --- |
| 50172 | Astafyeva, Natalya | Ethics Committee at the State Budgetary Educational Institution of Higher Professional Education  «Saratov State Medical University named after V.I. Razumovskiy» of Ministry of Health of Russian Federation  Chairperson: Dr. T.G. Kamenskikh | Kazan | Russia |
| 50173 | Starovoytova, Elena | Ethics Committee at State Budgetary Educational Institution Of Higher  Professional Education Siberian State Medical University of Ministry of Health of Russian Federation  Chairperson: Prof. E.B Bukreeva | Tomsk | Russia |
| 50174 | Yakusevich, Vladimir | Ethics Committee at the State Institution of Health Clinical Hospital for Emergency Care named after N.V. Solovyov  Chairperson: Mr. V.N Berezin | Yaroslavl | Russia |

| **Center number** | **Investigator** | **IEC/IRB name Committee chairperson**a **Assurance number** | **Address** | **Country** |
| --- | --- | --- | --- | --- |
| 50175 | Shaporova, Natalya | Ethics Committee at Academician I.P.Pavlov First Saint-Petersburg State Medical University  Chairperson: Prof. O.D. Yagmurov  Assurance Number : N/A | Saint-Petersburg | Russia |
| 50177 | Mihailov, Sergey | Independent Ethics Committee at Non-state Institution of Health  «Central Clinical Hospital #1 of  Open Joint Stock Company  «Russian Railways» Chairperson: Prof. A.A. Dmitriev  Assurance Number : N/A | Moscow | Russia |
| 50178 | Emelyanov, Alexander | Ethics Committee at Saint- Petersburg State Budgetary Institution of Health "City Out- Patient Hospital #94 of Nevsky District"  Chairperson: Mr. I.A. Kapushova  Assurance Number : N/A | Saint-Petersburg | Russia |
| 50179 | Fassakhov, Rustem | Local Ethics Committee at the Federal Budgetary Institution of Science «Kazan Research Institute of Epidemiology and Microbiology» of Federal Service for Supervision over Consumer Rights Protection and Human Welfare  Chairperson: Mr. S.V. Andreev  Assurance Number : N/A | Kazan | Russia |

| **Center number** | **Investigator** | **IEC/IRB name Committee chairperson**a **Assurance number** | **Address** | **Country** |
| --- | --- | --- | --- | --- |
| 31054  31053  31051  31056 | Roger, Albert Antepara, Ignacio Alonso, Tejedor Tabar, Ana Isabel | CEIC Hospital Germans Trias i  Pujol  Chairperson: Dr. Joan Costa  Pagés  Assurance Number : N/A | Barcelona | Spain |
| 42011 | Bjermer, Leif | Regional Ethical Review Board in Lund  Chairperson: Peter Kristiansson  Assurance Number : N/A | Lund | Sweden |
| 34028  34022  34024  34029 | Dr David Simcock *(previously Prof Neil Barnes)*  Prof Brian Lipworth  Dr Mark Blagden  Dr Michael Butler | NRES Committee London – Hampstead  Chairperson:  Assurance Number : N/A | Manchester | United  Kingdom |

a If available, the name of the committee chairperson at the start of the study is provided. IEC=Independent Ethics Committee; IRB=Institutional Review Board.
